# Long-term survival following hepatectomy, radiation, and chemotherapy for recurrent pancreatic carcinoma: a case report

**DOI:** 10.1186/s12957-017-1232-2

**Published:** 2017-08-23

**Authors:** Shigeru Fujisaki, Motoi Takashina, Ryouichi Tomita, Kenichi Sakurai, Tadatoshi Takayama

**Affiliations:** 1 Department of Surgery, Fujisaki Hospital, 1-25-11, Minamisuna, Kotoh-ku, Tokyo 136-0076 Japan; 2 Department of Surgery, Nippon Dental University School of life Dentistry, 2-3-16 Fujimi, Chiyoda-ku, Tokyo 102-8158 Japan; 3 Divisions of Breast and Endocrine Surgery, Nihon University School of Medicine, 30-1, Oyaguchikamimachi, Itabashi-ku, Tokyo 173-8610 Japan; 4 Digestive Surgery, Nihon University School of Medicine, 30-1, Oyaguchikamimachi, Itabashi-ku, Tokyo 173-8610 Japan

**Keywords:** Recurrent pancreatic carcinoma, Hepatectomy, Radiation, Chemotherapy

## Abstract

**Background:**

Recurrent pancreatic carcinoma (PC) is generally well known to have a poor prognosis. Cases in which multidisciplinary treatments have been remarkably effective are rare.

**Case presentation:**

Herein, we reported a case of long-term survival following a combination of hepatectomy for a liver metastasis and radiation and chemotherapy for abdominal lymph node metastases after a curative pancreaticoduodenectomy for PC. A 51-year-old Japanese man underwent a pancreaticoduodenectomy following a PC diagnosis in December 2011. After the surgery, the patient received 16 cycles of gemcitabine (GEM) adjuvant chemotherapy. Abdominal computed tomography (CT) after therapy with GEM (17 months after surgery) revealed a 1-cm nodule in the liver, for which the patient underwent partial hepatectomy in May 2013. Approximately 1 month after the hepatectomy, the patient underwent adjuvant chemotherapy using tegafur/gimeracil/oteracil (S-1) for 12 months. Approximately 1 year after the second surgery, an abdominal CT scan detected the abdominal lymph node metastases, for which the patient underwent radiation therapy. After the radiation therapy, combination therapy with 5-fluorouracil(5-FU)/leucovorin plus oxaliplatin or irinotecan was started in September 2014; 59 cycles of this chemotherapy have been administered up to the time of this report. At 67 months after the pancreaticoduodenectomy and 50 months after the hepatectomy, the patient has remained healthy with no relapse or recurrent lesions.

**Conclusion:**

We have managed a long-term survivor who underwent hepatectomy for liver metastasis and radiation therapy and chemotherapy for abdominal lymph node metastases after curative pancreaticoduodenectomy for PC.

## Background

Recurrent pancreatic carcinoma (PC) is generally well known to have a poor prognosis, although variety of modalities, including chemotherapy, have been used to treat recurrences. Because the progression of PC recurrence is generally extremely rapid, multiple lesions often exist at the time of recurrence detection. Cases in which multidisciplinary treatments have been remarkably effective are rare.

We herein report a case of recurrent PC in which this combination of therapies have been extremely successful. Specifically, the patient underwent hepatectomy for liver metastasis, followed by radiation and chemotherapy for abdominal lymph node metastases.

## Case presentation

A 51-year-old Japanese man underwent a subtotal stomach-preserving pancreaticoduodenectomy following the diagnosis of PC in December 2011. Histopathologic examination of the resected specimen revealed invasive ductal carcinoma; this PC case was classified as T2N1M0, stage IIB using the American Joint Committee on Cancer (AJCC) staging guidelines.

The patient demonstrated a relatively good postoperative course and was discharged from our hospital in remission 26 days after the surgery. Thereafter, the patient underwent adjuvant gemcitabine (GEM) chemotherapy at 1000 mg/m^2^/day on days 1, 8, and 15 during a 28-day cycle.

**Fig. 1:**
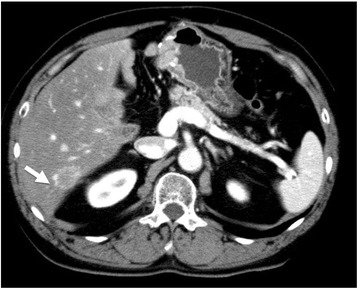
Abdominal computed tomography scan shows liver metastasis in segment 6 (arrow)

After 16 cycles of GEM or 17 months after surgery, an abdominal computed tomography (CT) scan revealed a 1-cm nodule in the liver (Fig. 1). Review of the abdominal CT scan that was performed 4 months previous revealed obscure hints of a nodule in the same location. Notably, even after 4 months of observation, there was only one liver metastasis, and no other distant metastases were observed.

We performed a partial hepatectomy (segment 6) in May 2013. The patient demonstrated a relatively good postoperative course. One month after the hepatectomy, he received adjuvant chemotherapy with tegafur/gimeracil/oteracil (S-1) for 12 months.

**Fig. 2:**
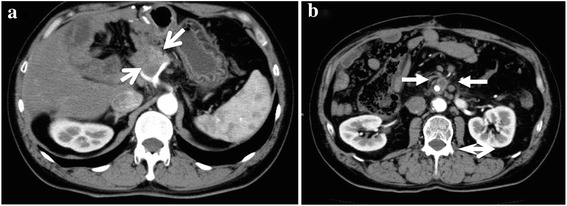
About 1 year after the hepatectomy, the abdominal lymph node metastases around the celiac artery (**a**; arrow) and along the superior mesenteric artery (**b**; arrow) are detected by the abdominal computed tomography

Approximately 1 year after the second surgery, repeat abdominal CT scan detected abdominal lymph node metastases around the celiac artery and along the superior mesenteric artery (Fig. 2). Because the patient had already received GEM and S-1, we initially treated the lymph node metastases with radiation therapy alone.

The macroscopically visible tumors as well as the locoregional lymph nodes were irradiated with an International Commission on Radiation Units and Measurements (ICRU) reference dose of up to 39.6 Gy, which was applied in 22 fractions of 1.8 Gy/day.

Because the administration of oxaliplatin and irinotecan for PC was approved for use in Japan after December 2013, we decided to add sequential chemotherapy. In particular, the combination therapy of 5-FU/leucovorin plus oxaliplatin or irinotecan was given initially in September 2014. Because the patient had severe fatty liver with a poor hepatic function reserve (the ICG-R15 was 46.0%), irinotecan was initially excluded. After receiving 10 cycles of treatment, the peripheral neuropathy of the patient worsened (grade 2). Consequently, oxaliplatin was withdrawn from the regimen, and irinotecan was administered from March 2015.

**Fig. 3:**
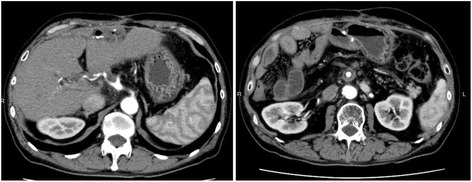
The abdominal computed tomography scan in April 2016, about 20 months after the radiation therapy, shows no relapse of the recurrent lesions

**Fig. 4:**
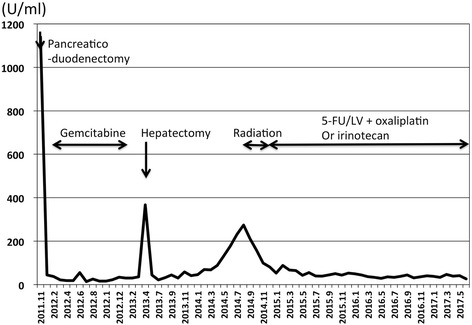
Value of CA19–9 (U/ml)

From September 2014 to the present, a total of 59 cycles of chemotherapy have been administered. Even at 67 months after the pancreaticoduodenectomy and 50 months after hepatectomy, the patient has remained healthy with no relapse of recurrent lesions (Fig. 3), and the value of serum CA19–9 has been within the normal range (Fig. 4).

## Discussion

The prognosis of PC is very poor, particularly when metastasis has occurred. Re-resection of PC relapse has only been reported only as single case reports or in small series [1–11]. Sperti et al. reviewed recent works [12] concerning repeat surgery for local recurrence [1, 2, 5–10] (*n* = 62; lymph node metastases, tumor relapse in the bed of the pancreatic resection, and tumor recurrence in the pancreatic remnant) and metastatic recurrence [1, 5, 10, 11] (*n* = 37). The median survival was 17.5 months for the cases with local recurrence and 18.6 months for those with metastatic recurrence.

On the other hand, limited information is available regarding the importance of chemoradiation applied in local or distant recurrence of PC [12]. Although chemoradiotherapy has been mentioned as an effective option in several reports, it has brought limited benefit, and has not brought a dramatic improvement in survival, compared with re-operation after recurrence. In the limited cases wherein resection of recurrent lesions is feasible, surgery is supposed to play a central role among the choices of multimodality treatment for improvement of prognosis.

The effectiveness of hepatectomy for liver metastases has been confirmed in colorectal cancer. However, the appropriate indication for hepatectomy to treat liver metastases from PC has not been established. There have been few reports that have indicated the criteria for resection of hepatic metastases from PC. According to a study from our institute [13], chest and abdominal CT examinations after a radical resection of intractable hepatobiliary pancreatic cancers should be performed approximately three times a year; as we have been doing, attention should be focused on the emergence of small nodules in the liver. Instead, performing hepatectomy immediately after detecting a small nodule, we typically observe the patient for 3–6 months. If a single nodule is enlarging but has not spread to other organs, we may decide to perform hepatectomy. However, when the detected nodule is single and is larger than 1 cm in size, we consider performing the hepatectomy immediately.

In the present case, review of the abdominal CT scan taken 4 months prior to the detection of the liver nodule revealed obscure hints of a nodule at the same location. Fortunately, even after 4 months of observation, there remained only one liver metastasis, and no other organ metastases were observed. Therefore, we decided to perform a partial hepatectomy. After the hepatectomy, there have been no additional liver metastases to date, but another recurrent site in the abdominal lymph nodes was detected approximately 1 year after.

Chemoradiotherapy is considered to be an effective treatment option in those patients who present with local metastasis after primary surgery for PC [14]. However, in the present case, adjuvant chemotherapy (GEM and S-1) had already been administered. Therefore, only radiation therapy was initially performed for the lymph node metastases.

Since December 2013, the administration of oxaliplatin and irinotecan as a combination of anticancer agents referred to as FOLFIRINOX has been approved in Japan. This chemotherapy has adverse toxicities [15]. In this case, the patient had severe fatty liver with a poor hepatic functional reserve (ICG-R15 was 46.0%); therefore, irinotecan was excluded from the regimen. After receiving 10 cycles, the patient experienced worsening peripheral neuropathy (grade 2). Therefore, we then discontinued oxaliplatin and added irinotecan. Without any subsequent liver dysfunction, we have been able to continue the combination therapy of 5-FU/leucovorin plus irinotecan.

We had experienced a case that suggested that multimodality management of recurrent PC might lead to better survival and quality of life. More studies are needed to define the clinical outcomes of resection in combination with other therapeutic modalities for the metastases.

## Conclusions

We have managed a long-term survivor who underwent hepatectomy for liver metastasis and radiation therapy and chemotherapy for abdominal lymph node metastases after a curative pancreaticoduodenectomy for PC. It is generally difficult to treat recurrent PC. Our experience on this case that suggested that multimodality management of recurrent PC might lead to better survival and quality of life. There is a need for further studies to evaluate the treatment strategies for metastases, including the indications of resection for the metastases in combination with other therapeutic modalities.

## Abbreviations

5-FU: 5-Fluorouracil
AJCC: The American Joint Committee on Cancer
CT: Computed tomography
GEM: Gemcitabine
ICG-R15: Indocyanine green retention test 15
PC: Pancreatic carcinoma
S-1: Tegafur/gimeracil/oteracil
